# Student mental wellbeing as an interconnected system: positioning academic, social, and coping processes

**DOI:** 10.1016/j.ssmph.2026.101936

**Published:** 2026-06-08

**Authors:** Sybren Slimmen, Olaf Timmermans, Lilian Lechner, Peter Verboon, Anke Oenema

**Affiliations:** a Department of Health Psychology, Open University, Mailbox 2960, 6401 DL, Heerlen, the Netherlands; b Research Group Healthy Region, HZ University of Applied Sciences, Edisonweg 4, 4382 NW, Vlissingen, the Netherlands; c Centre for Research and Innovation in Care, Antwerp University, Universiteitsplein 1, 2610, Wilrijk, Belgium

**Keywords:** Student mental wellbeing, Network analysis, Academic stressors, Coping styles, Social environmental factors, Longitudinal associations

## Abstract

Student mental wellbeing has become a growing concern in higher education, with increasing numbers of students reporting stress and psychological difficulties. Although academic factors, social environmental factors, and coping styles are all considered relevant, less is known about how these factors are interconnected in relation to mental wellbeing within a broader system. This study applied a network approach to examine how these factors were interconnected at the start of the academic year and how they were longitudinally associated later in the academic year.

Survey data were collected among students at a Dutch university of applied sciences in November 2023 (T1; N = 497) and June 2024 (T2; n = 207). Data were collected on mental wellbeing, academic stressors, academic self-perceptions, coping styles and social environmental factors. A Gaussian Graphical Model was used to estimate the cross-sectional network at T1, and longitudinal associations from T1 to T2 were examined using regularized regression (LASSO).

The findings showed that associations in the cross-sectional network were generally modest. Connections were mainly observed between conceptually related variables, including academic stressors, coping styles, and social environmental factors. Mental wellbeing showed few direct associations with the other constructs in the network, but both mental wellbeing and social support showed relatively high centrality, indicating that they were strongly connected within the overall network structure. Longitudinal analyses indicated substantial stability across the academic year, as most variables were primarily related to their own prior levels and only a limited number of associations between different variables were observed over time.

These findings suggest that student mental wellbeing was embedded in a relatively stable system of related experiences. They also illustrate that cross-sectional variable network approaches may have limitations when conditional associations are weak and constructs overlap conceptually, particularly when used to draw conclusions about complex socio-ecological processes.

## Highlights

• Mental wellbeing and social support had the most central position in the network.
• Most associations in the network were observed within clusters of related variables.
• Mental wellbeing and related variables showed stability across the academic year.

## 1 Introduction

Concerns about student mental wellbeing have grown in recent years, as a substantial proportion of students report mental health difficulties throughout their studies (Hill et al., 2024; Roy et al., 2025). Increasingly, these difficulties are recognised as part of a broader public health challenge affecting student populations worldwide (Sheldon et al., 2021). Evidence from multiple countries and educational settings consistently points to elevated levels of stress, reduced mental wellbeing, anxiety, and depressive symptoms among students, underscoring the widespread nature of these concerns (Kirkbride et al., 2024; Roy et al., 2025; Slimmen et al., 2022; Versteeg & Kappe, 2021). Understanding underlying factors that drive or undermine mental wellbeing is crucial in designing prevention programmes. At the same time, existing evidence suggests that mental wellbeing cannot be attributed to single causes, but emerges from the dynamic interaction between multiple aspects of students’ daily lives (Langellier et al., 2019; Rod et al., 2023; Stronks et al., 2025). Existing research has often concentrated on individual-level determinants and immediate environmental influences on mental wellbeing, however, there is consensus that increasing attention needs to be given to the role of broader contextual conditions in shaping mental wellbeing (Raad voor Volksgezondheid & Samenleving, 2025; Verhoog et al., 2024). Addressing interconnected influences on student mental wellbeing calls for a systemic and integrative perspective that goes beyond isolated determinants.

Student mental wellbeing encompasses psychological functioning, life satisfaction, the maintenance of meaningful relationships, and also captures positive functioning, resilience, and experiences of thriving across multiple life domains (Barkham et al., 2019; Tennant et al., 2007). It can be understood as a complex and dynamic phenomenon shaped by the interplay of multiple influences (Scherer & Leshner, 2021). From a socio-ecological perspective, student mental wellbeing can be understood as the result of interacting influences across multiple levels, including micro-, meso-, and macro-level contexts (Bronfenbrenner, 1994). Within this broader system, the academic context, coping styles, and social environmental factors can be understood as interconnected components that exert substantial influence on student mental wellbeing (Slimmen et al., 2024, 2025). These influences operate across interconnected environmental levels, ranging from students’ day-to-day academic experiences and coping responses to wider institutional and community contexts (Bronfenbrenner, 1994; Ungar & Theron, 2020). Within this system, academic factors such as perceived workload, performance pressure, and academic self-perceptions may interact with how students cope with stressors, while social environmental factors provide conditions that can either buffer or intensify these processes (Butler et al., 2022; Chaudhry et al., 2024; Perry et al., 2024; Sheldon et al., 2021). Moreno-Montero et al. (2024) show that coping styles influence student mental wellbeing by shaping how students respond to stressors, with adaptive strategies supporting wellbeing and avoidant strategies undermining it. Their findings further suggest that the role of coping styles cannot be understood in isolation, but rather in relation to other personal and contextual resources. Students who are more strongly embedded in their social environments, for example through peer relationships or academic networks, tend to be better equipped to cope with the psychological challenges associated with their studies (Pointon-Haas et al., 2024). Although the relevance of academic factors, coping styles, and social environmental factors has been well documented, less is known about how these factors are interconnected and how they affect student mental wellbeing within a network (Kirkbride et al., 2024; Slimmen et al., 2022; Stronks et al., 2025).

To better capture the complexity of factors related to student mental wellbeing, network analysis has increasingly been used as an alternative to traditional variable-centred approaches (den Bleijker et al., 2025). Rather than examining isolated predictors and outcomes, a network approach conceptualizes mental wellbeing as part of a system of interrelated variables, in which associations between factors are explicitly modelled (Epskamp, Borsboom, & Fried, 2018; Hevey, 2018). In such models, variables are represented as nodes and their conditional associations as edges, allowing insight into how different factors co-occur and interact within a broader structure. This perspective is particularly relevant when multiple factors from the academic context, social environment, and coping styles are expected to influence one another simultaneously, as is the case for student mental wellbeing (Segú-Odriozola, 2025; Slimmen et al., 2025). By examining both the structure of associations and the relative importance of variables within the network, network analysis provides a descriptive framework for identifying which factors are more centrally embedded in the system, without imposing assumptions about linear causality. A network approach is therefore relevant to a socio-ecological perspective on student mental wellbeing because it allows constructs from different levels to be examined simultaneously within one empirical structure. It can provide insight into whether variables that are conceptually positioned at similar levels, or within similar domains, are more strongly interconnected than variables positioned across different levels. At the same time, network analysis does not directly model the hierarchical or nested structure that is central to socio-ecological theory.

The present study uses network analysis in an exploratory way, to examine how academic factors, social environmental factors, and coping styles are interconnected in relation to student mental wellbeing. These factors are deliberately drawn from different levels of the socio-ecological model to explore their relative positioning and interconnections within the broader system related to student mental wellbeing. Coping styles and self-perceptions were considered more proximal-level (individual-level) constructs, as they reflect students' own responses to stressors and perceptions of their academic abilities. Academic stressors, social support and social network satisfaction were positioned at the micro-level, as they concern students' immediate relational and educational environment. Social cohesion, community integration, community organizations, and community participation were considered meso-level constructs, as they reflect students' perceived connection with the wider educational and institutional community. From a socio-ecological perspective, proximal related constructs, may be expected to show more direct associations with mental wellbeing, whereas broader meso-level constructs may be more diffusely connected through students’ immediate social and academic experiences. The first objective is to estimate a cross-sectional network at the start of the academic year (T1) in order to describe the pattern of associations between these factors and to identify which variables occupy a more central position in the network surrounding mental wellbeing. The second objective is to examine longitudinal associations between variables measured at T1 and T2, providing insight into how academic factors, social environmental factors, coping styles, and mental wellbeing assessed at the first time point are related to these variables later in the academic year. By offering a system-oriented overview of how these factors are interconnected and positioned within the network around mental wellbeing, the findings can support educational institutions and student support services to determine which factors are most influential in a network and could be targeted in interventions to support student mental wellbeing.

## 2 Methods

### 2.1 Study design

This study employed a prospective longitudinal quantitative design with two waves of survey-based data collection. It is part of a broader project examining how academic, social, and environmental factors contribute to student mental wellbeing. The first wave of data collection, conducted in November 2023, included a wide range of constructs. For the present study, the following variables measured at T1 were used: mental wellbeing, academic stressors, academic self-perceptions, coping strategies, social support, social network satisfaction, social cohesion, community integration, community organizations and community participation. The second wave (T2), administered in June 2024, reassessed mental wellbeing, academic stressors, academic self-perceptions, coping strategies, and social network satisfaction, allowing examination of both the cross-sectional structure at T1 and longitudinal developments across time. The study was preregistered in the Open Science Framework (OSF; https://doi.org/10.17605/OSF.IO/BUCRM) and received ethical approval from the Open University ethical review board (CETO_RP459/U202309407).

### 2.2 Participants and procedure

The study population comprised students enrolled at a University of Applied Sciences in the Netherlands, which offers bachelor's programmes across three domains: Business, Vitality and Hospitality; Health, Education and Wellbeing; and Technology, Water and Energy. Within the Dutch higher education system, universities of applied sciences provide higher professional education (*hoger beroepsonderwijs*, HBO) and offer professionally oriented bachelor's programmes that prepare students for occupational fields, such as nursing, social work, education, engineering, and business, with curricula typically combining theoretical education, applied learning, internships, and practice-based research. The institution has two campuses, and serves a regional, national, and international student population. The institution serves approximately 5000 students. At the time of data collection, approximately 4400 full-time students were enrolled and invited to participate. These full-time students formed the source population for the present analyses. Data collection took place online through a GDPR-compliant survey platform using validated questionnaires available in Dutch and English, allowing students to choose their preferred language. Validated versions of the instruments were used in both languages, and adapted items were reviewed by the research team for conceptual equivalence. In November 2023 (T1), all enrolled students received an email invitation to take part voluntarily and were offered the chance to enter a raffle for Apple AirPods. After providing informed consent, students completed the online questionnaire. The second measurement wave in June 2024 (T2) included a shorter follow-up. Only students who participated in the first wave were approached again for the second wave. Both data collection periods remained open for three weeks, during which students received two reminder emails.

### 2.3 Instrument and measured concepts

Mental wellbeing was measured using the Warwick–Edinburgh Mental Wellbeing Scale (Tennant et al., 2007), which captures mental wellbeing through positive functioning and emotional wellbeing through 14 items rated on a 5-point Likert scale. Academic stressors and academic self-perception were assessed with the 18-item Perceptions of Academic Stress questionnaire (Bedewy & Gabriel, 2015), measuring workload, performance pressure, time restraints (e.g. perceived lack of sufficient time to meet academic demands), and confidence in academic ability using 5-point Likert responses. Coping styles were measured with the 28-item Brief COPE (Muller & Spitz, 2003), which distinguishes avoidant, problem-focused, and emotion-focused coping on a 4-point Likert scale. Social environmental factors were operationalized using five distinct variables. Social network satisfaction was assessed using the three-item CEL Loneliness Scale (Goossens & Beyers, 2002), with responses rated on a 4-point Likert scale. Community integration, community organizations, and community participation were measured using validated subscales from the Perceived Community Support Scale (Herrero & Gracia, 2007), assessing perceived support from institutional groups rated with 14 items on a 5-point Likert scale. Social cohesion within the educational community was assessed using eight adapted items from the Perceived Neighbourhood Social Cohesion Scale (Dupuis et al., 2017), covering three dimensions: trust, attachment, and tolerance/respect. The eight items used in the present study were selected because they captured aspects of social cohesion that were conceptually relevant and transferable to an educational community, including connectedness, trust, reliability, mutual support, attentiveness to others, friendliness, and respectful interaction. The adaptation involved replacing references to the neighbourhood with references to the educational community, while preserving the conceptual focus of the original items. Social support was measured with the 12-item Multidimensional Scale of Perceived Social Support (Zimet et al., 1988), capturing support from family, friends, and significant others on a 7-point Likert scale. Table 1 provides an overview of all constructs, instruments with internal consistency, response formats, and example items.

**Table 1:** Study variables and corresponding measurement instruments.

Concept	Instrument	α	Response categories	Scale	Number of items	Example item	Occasion
Mental wellbeing	Warwick–Edinburgh Mental Wellbeing Scale (Tennant et al., 2007)	0.91	5-point Likert	14-70	14	*“I've been feeling optimistic about the future.”*	T1/T2
Academic stressors	Perceptions of Academic Stress (Bedewy & Gabriel, 2015)	0.89	5-point Likert	1-5	18	*“I am confident that I will be a successful student.”*	T1/T2
Coping styles	Brief COPE (Muller & Spitz, 2003)	0.77	4-point Likert	1-5	28	*“I've been taking action to try to make the situation better.”*	T1/T2
Social network satisfaction	CEL Loneliness Scale (Goossens & Beyers, 2002) – satisfaction items	0.79	4-point Likert	3-12	3	*“I am content with my friendships and relationships.”*	T1/T2
Social cohesion	Perceived Neighbourhood Social Cohesion Scale (Dupuis et al., 2017)	0.80	5-point Likert	1-5	8	*“People at this university treat each other with respect.”*	T1
Community integration	Perceived Community Support Scale (Herrero & Gracia, 2007) – subscale	0.88	5-point Likert	1-5	4	*“I identify with my university.”*	T1
Community organizations	Perceived Community Support Scale (Herrero & Gracia, 2007) – subscale	0.85	5-point Likert	1-5	5	*“I would find a source of satisfaction for myself.”*	T1
Community participation	Perceived Community Support Scale (Herrero & Gracia, 2007) – subscale	0.82	5-point Likert	1-5	5	*“I take part in social activities in my community.”*	T1
Social support	Multidimensional Scale of Perceived Social Support (Zimet et al., 1988)	0.90	7-point Likert	1-7	12	*“My friends really try to help me.”*	T1

### 2.4 Analysis

Prior to the main analyses, the data were screened for distributional issues, missing data, and the internal consistency of multi-item scales using Cronbach's alpha. Skewness values were inspected as part of the preliminary data screening and indicated no substantial distributional deviations, allowing all variables to be analysed in their original metric while preserving the interpretability of the scale scores. Missing data were examined at the variable level. Missing values were handled using pairwise exclusion. Descriptive statistics were conducted to summarize means, standard deviations, and demographic characteristics of the sample. An attrition analysis was conducted to examine whether students who participated at T2 differed from those who did not on baseline study variables.

To examine associations between the study variables, we conducted a network analysis using a Gaussian Graphical Model (GGM) based on cross-sectional data collected at T1. In this network, each variable is represented as a node, and connections between nodes (edges) represent regularized partial correlations. This means that the association between two variables is estimated while taking into account all other variables in the network, providing insight into their unique relationship (Epskamp, Waldorp, et al., 2018). The planned T1 network included 14 nodes. Because sample size requirements for network analysis depend on the expected network structure, edge strength, density, and desired precision, no fixed minimum sample size rule was applied. As the analysis was exploratory, regularization and bootstrap procedures were used to support cautious interpretation of the estimated network (Constantin et al., 2023).

The network was estimated using graphical LASSO with EBIC model selection (EBICglasso) and the default tuning parameter γ = 0.50. This method reduces very small partial correlations to zero, effectively removing weak associations from the network. As a result, the final network is more simplified and easier to interpret, with a focus on the strongest and most meaningful associations between variables (Hevey, 2018). Because the aim of the cross-sectional network analysis was to explore the relative positioning of individual constructs within the network, node-level centrality indices were selected as the most appropriate measures for this study. These indices provide information on how strongly or strategically a variable is embedded within the overall network and can therefore help identify variables that may be relevant from a practical or intervention-oriented perspective. Strength indicates how strongly a variable is connected to other variables overall, regardless of whether these associations are positive or negative. Expected influence also reflects overall connectedness, but takes the direction of associations into account and therefore shows whether a variable is mainly connected through positive or negative associations. Closeness reflects how near a variable is to all other variables in the network, indicating whether it is positioned close to the broader system of variables. Betweenness indicates whether a variable lies on the shortest paths between other variables, and may therefore reflect a potential bridging position within the network (Epskamp, Borsboom, & Fried, 2018). Following best-practice guidelines (Hevey, 2018), the accuracy and stability of the network were assessed using bootstrap procedures. A detailed description of the bootstrap analysis is provided in the supplementary file. Following recommendations by Epskamp, Borsboom, and Fried (2018), only centrality metrics with a correlation-stability coefficient above 0.25 were considered sufficiently stable for interpretation. All analyses were performed in JASP (Network module; JASP Team), using default settings (Love et al., 2019).

To examine how variables were related over time, we used a regression approach called the Least Absolute Shrinkage and Selection Operator (LASSO), implemented in R (RStudio) with the glmnet package (Hastie & Qian, 2014, pp. 1–30). This method is useful when many variables are included that may be related to each other, as it reduces the risk of overfitting and helps identify the most important associations by shrinking small regression coefficients exactly to zero (Hastie & Qian, 2014, pp. 1–30; McNeish, 2015). In this analysis, all variables measured at T1 were entered simultaneously to examine how they were associated with variables at T2, both within the same variables over time and across different variables. The LASSO method applies a penalty (ℓ_1_ constraint) that shrinks weaker associations to zero, resulting in a simpler model that retains only the most relevant relationships. Regression coefficients that remained non-zero were interpreted as longitudinal associations between variables over time. Following recommendations by van Erp et al. (2019), the results were interpreted descriptively, focusing on the associations that remained after this selection process and excluding weaker relationships. Because LASSO coefficients are penalized estimates and were interpreted descriptively, conventional confidence intervals were not calculated. A detailed description of this procedure is provided in the supplementary file.

## 3 Results

### 3.1 Participants

At the first measurement occasion in November 2023 (T1), 497 students participated in the study. For 384 students, complete data were available for the cross-sectional network analysis; for the remaining students, one or more values were missing and were handled using pairwise exclusion. At the second measurement occasion in June 2024 (T2), 207 students had complete data for the longitudinal analysis (Table 2). The average age of participants was similar at both time points. The distribution of gender remained comparable across T1 and T2, with most participants identifying as female, followed by male, and a small proportion identifying as other. Students represented three educational domains, with the largest proportion enrolled in Health, Education and Wellbeing at both measurement occasions. Participants were drawn from all years of study, with first- and second-year students constituting the largest groups. Attrition analyses comparing students who participated in both waves (n = 207) with those who participated only at T1 (n = 290) revealed no significant differences in age, t(493) = −1.215, p = .225, mental wellbeing at T1, t(495) = −0.759, p = .448, gender, χ^2^(1) = 0.104, p = .747, or international student status, χ^2^(1) = 3.13, p = .077.

**Table 2:** Demographic characteristics of the study sample at T1 and T2.

	T1	T2
**Respondents (N)**	497	207
**Age (Mean)**	20,54 (SD 2,86)	20,35 (SD 2,77)
**Gender**		
Female	61,8% (N = 307)	62,3% (N = 129)
Male	37,2% (N = 185)	36,2% (N = 75)
Other	<1,0% (N = 5)	1,5% (N = 3)
**Domain of education**		
Business, Vitality and Hospitality	26,0% (N = 129)	21,3% (N = 44)
Health, Education and Wellbeing	40,8% (N = 203)	38,6% (N = 80)
Technology, Water and Energy	33,2% (N = 165)	40,1% (N = 83)
**Year of study**		
1	33,0% (N = 164)	35,7% (N = 74)
2	27,6% (N = 137)	30,4% (N = 63)
3	12,9% (N = 64)	11,6% (N = 24)
4	21,5% (N = 107)	18,4% (N = 38)\
+	5,0% (N = 25)	3,9% (N = 8)
**Location**		
Vlissingen	72,2% (N = 359)	65,7% (N = 136)
Middelburg	27,8% (N = 138)	34,3% (N = 71)
**Type student**		
Domestic	78,3% (N = 389)	74,4% (N = 154)
International	21,7% (N = 108)	25,6% (N = 53)

***Note.*** The category “Other” combines students who identified as non-binary and students who preferred not to disclose their gender; these categories were combined due to the small number of responses.

### 3.2 Mean scores of variables

At T1, mental wellbeing showed a moderate level on the 14–70 scale, while social network satisfaction and perceived social support were relatively high in relation to their respective scale ranges. Social cohesion, community integration, community organizations, and community participation showed scores around the midpoint of their 1–5 scales. Coping styles and academic stressors were generally reported at moderate levels.

At T2, mental wellbeing again reflected a moderate level, and social network satisfaction remained relatively high. Coping styles and academic stressors remained broadly comparable to T1, with scores again situated around the midpoint of their scales. Full descriptive statistics for all variables at T1 and T2 are presented in Table 3.

**Table 3:** Mean scores of variables at T1 and T2.

Variable	T1 M (SD)	T2 M (SD)
Mental wellbeing	46.73 (8.23)	47.10 (7.47)
*Academic stressors*		
Pressure to perform	2.73 (0.70)	2.66 (0.71)
Perceptions of workload	2.78 (0.68)	2.78 (0.71)
Academic self-perceptions	3.37 (0.80)	3.46 (0.74)
Time restraints	2.65 (0.65)	2.59 (0.62)
*Coping styles*		
Avoidant coping	2.10 (0.50)	2.10 (0.55)
Problem-focused coping	2.94 (0.55)	3.04 (0.49)
Emotion-focused coping	2.84 (0.44)	2.86 (0.46)
Social network satisfaction	8.85 (2.13)	8.94 (1.98)
Social cohesion	3.55 (0.56)	—
Community integration	2.75 (0.70)	—
Community organizations	3.40 (0.80)	—
Community participation	2.62 (0.91)	—
Social support	5.36 (1.18)	—

### 3.3 Network analysis

**Fig. 1:**
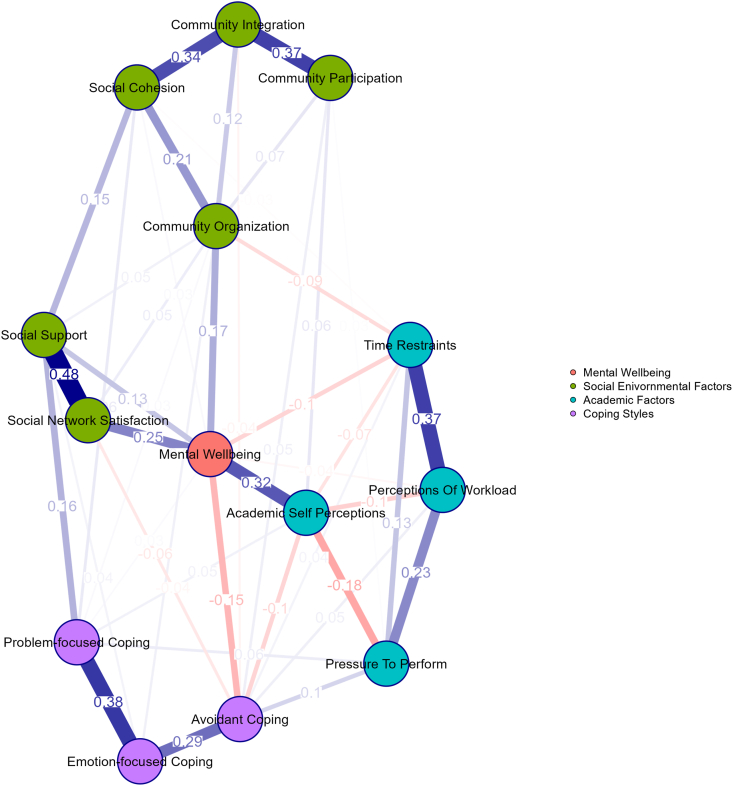
Graphical representation of the estimated Gaussian graphical model based on T1 variables ***Note.*** Nodes represent mental wellbeing, social environmental factors, academic factors, and coping styles. Edges represent regularized partial correlations; blue edges indicate positive and red edges negative associations, with thickness reflecting absolute edge weight.

The estimated Gaussian Graphical Model at T1 is presented in Fig. 1. The figure shows the regularized conditional associations estimated in the original sample. Nodes represent the study variables, and edges represent partial correlations after controlling for all other variables in the network. Edge thickness reflects the relative strength of the estimated association in this sample: thicker lines indicate stronger estimated associations, while thinner lines indicate weaker estimated associations. Fig. 1 should therefore be read as a descriptive map of the estimated network structure, not as evidence that each individual edge is equally precise or stable.

In the network estimated form the observed data, the clearest positive conditional associations involving mental wellbeing were observed with academic self-perceptions and social network satisfaction. Within the social environmental variables, positive associations were observed between social support and social network satisfaction, and between community integration and both social cohesion and community participation. Within the academic variables, perceptions of workload were positively associated with time restraints. Emotion-focused coping was positively associated with both problem-focused coping and avoidant coping. Negative conditional associations were generally smaller in magnitude. Mental wellbeing was negatively associated with avoidant coping and time restraints, while avoidant coping and pressure to perform were negatively associated with academic self-perceptions. Academic self-perceptions were also negatively associated with perceptions of workload.

The bootstrap analysis was used to evaluate the uncertainty around these estimated edge weights. This distinction is important: Fig. 1 shows the estimated associations in the original sample, whereas the bootstrap confidence intervals, in Supplementary Figure A, show how precise or uncertain these estimates were. The bootstrap results indicated considerable uncertainty around several individual edges, with many confidence intervals overlapping zero. Therefore, the network was interpreted primarily in terms of the overall pattern of associations and the relative positioning of variables, rather than as strong evidence for specific individual edges.

### 3.4 Centrality indices

**Fig. 2:**
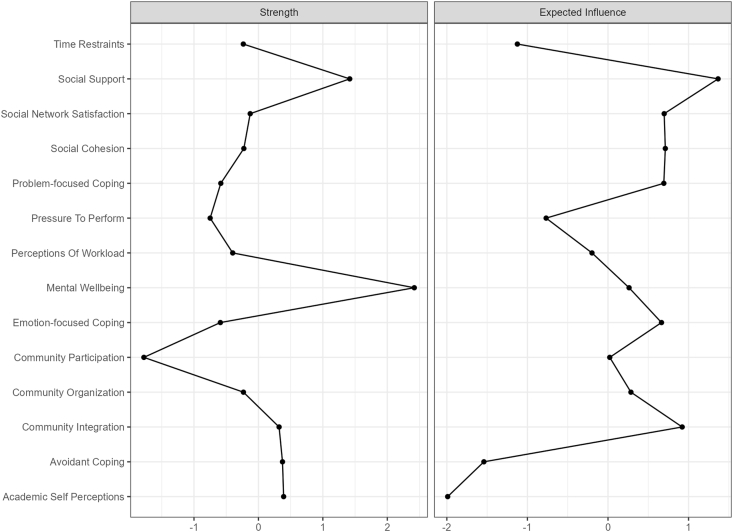
Centrality plots illustrating node strength and expected influence in the network depicted in Fig. 1 ***Note****.* Strength reflects the sum of the absolute values of the regularized partial correlations connected to each node, whereas expected influence reflects the sum of signed edge weights. Values are standardized, with higher absolute values indicating greater connectivity within the network.

Strength centrality indicated that mental wellbeing and social support were relatively more strongly connected to other variables in the network, whereas most other variables showed below-average strength (Fig. 2). Expected influence showed a different pattern, with social support and community integration displaying predominantly positive associations, while academic self-perceptions, avoidant coping, and time restraints showed predominantly negative associations. Coping variables showed a more heterogeneous pattern, with differences in the direction of associations across coping strategies. Node strength and expected influence were examined descriptively, whereas betweenness and closeness were not interpreted due to insufficient stability. Case-dropping bootstrap analyses showed that only strength reached a correlation-stability coefficient exceeding the recommended threshold of 0.25, while betweenness and closeness did not (Supplementary File – Figure B). For this reason, betweenness and closeness were not interpreted. Strength and expected influence are reported for descriptive purposes only. Additional insight into the contribution of individual edges to node strength is provided in the Supplementary File – Figure C, which presents a matrix visualization of non-zero edges retained after regularization and the corresponding node strength values.

### 3.5 Longitudinal associations across measurement occasions

Several variables showed clear autoregressive associations, indicating that scores at T1 were related to scores on the same constructs at T2. The strongest of these was observed for mental wellbeing (β = 0.44), followed by perceptions of workload (β = 0.37), pressure to perform (β = 0.36), academic self-perceptions (β = 0.36), and time restraints (β = 0.28). Autoregressive associations were also retained for social network satisfaction (β = 0.24) and for all three coping styles, with standardized coefficients ranging from β = 0.25 to β = 0.27.

A small number of associations between different variables were observed. The strongest of these concerned social network satisfaction at T2, which was related to social support at T1 (β = 0.20). Smaller associations were found between social network satisfaction at T2 and academic self-perceptions at T1 (β = 0.04), as well as mental wellbeing at T1 (β = 0.03). Mental wellbeing at T1 was also weakly related to academic self-perceptions at T2 (β = 0.01) and avoidant coping at T2 (β = −0.01). Time restraints at T1 showed small associations with academic self-perceptions at T2 (β = −0.04) and perceptions of workload at T2 (β = 0.01), while perceptions of workload at T1 was weakly related to time restraints at T2 (β = 0.01).

## 4 Discussion

The aim of this study was to examine how academic factors, social environmental factors, and coping styles are interconnected in relation to student mental wellbeing, using a network approach combined with a longitudinal perspective. By estimating a cross-sectional network at the start of the academic year and examining longitudinal associations across two measurement occasions, the study sought to move beyond isolated associations and provide insight into how multiple factors jointly relate to mental wellbeing over time. The findings are interpreted from a socio-ecological and complex systems perspective, in which mental wellbeing is understood as an outcome that emerges from interrelated academic, social, and individual processes rather than as the result of single determinants.

### 4.1 Network analysis

The network analysis showed that partial associations between variables were generally weak, and that only a limited number of variables were directly connected to mental wellbeing. This is notable, as previous research has consistently identified academic factors, coping styles, and social environmental factors as important in relation to student mental wellbeing (Campbell et al., 2022; Kirkbride et al., 2024; Liverpool et al., 2024; Roy et al., 2025; Slimmen et al., 2022; Versteeg & Kappe, 2021). The current findings suggest that, when considered simultaneously within a network of conditional associations, these factors do not show strong unique relationships with mental wellbeing. This divergence may indicate that previously reported associations partly reflect shared variance between closely related constructs, rather than independent effects of individual factors. Instead, the results of this study point towards a pattern in which associations are small, diffuse, and potentially shared across multiple interrelated variables.

One possible interpretation of this pattern, viewed through a socio-ecological lens, is that variables differ in their proximity to mental wellbeing within the system (Kilanowski, 2017). In the present network, only a small number of variables retained direct associations with mental wellbeing after regularization, whereas several variables—particularly those reflecting broader social or contextual conditions—were no longer directly connected. This may suggest that such factors are positioned more distally, in the sense that their associations with mental wellbeing are not unique when other variables are taken into account. This interpretation is consistent with longitudinal findings showing that meso-level social environmental factors are often related to mental wellbeing both directly and indirectly through more proximal mechanisms, such as social support and social network satisfaction, rather than through strong independent associations (Slimmen et al., 2025). Rather than indicating that these factors are unimportant, this pattern may reflect that their relevance is intertwined with other variables in the network.

Mental wellbeing and social support occupy the most central positions in the network, indicating that these are the variables most closely connected to other aspects of students’ experiences. In practical terms, this suggests that social support is one of the few factors that is directly linked to mental wellbeing and at the same time connected to other conceptually related variables, such as coping. Strengthening social support may therefore have broader relevance, as it is embedded in multiple parts of the system rather than operating in isolation. This is in line with previous research showing that social support plays a key role in buffering stress and supporting adaptive coping in students (Butler et al., 2022; Perry et al., 2024). Academic self-perceptions also occupies an important position in the estimated network, with predominantly negative associations with stress-related variables such as workload, pressure, and time restraints—factors consistently linked to poorer mental wellbeing in the literature (Bedewy & Gabriel, 2015; Roy et al., 2025; Slimmen et al., 2022). This suggests that more positive academic self-perceptions may act as an important counterbalance within these strain-related patterns.

The role of coping styles in the network was notable, primarily because coping was only weakly connected to mental wellbeing. This contrasts with previous research in which coping strategies have been described as relevant to how students manage mental health challenges and stressful life events (Liverpool et al., 2024). Emotion-focused coping was associated with both problem-focused coping and avoidant coping, suggesting that different coping styles may not function as entirely separate mechanisms, but may be interrelated within students’ broader coping responses. This is relevant because previous studies often distinguish problem-focused or approach-oriented coping as more constructive, whereas avoidant coping is more often associated with poorer mental health outcomes (Thomas et al., 2024). The present findings do not clarify the direction or meaning of these associations, but indicate that emotion-focused coping is connected to other forms of coping rather than standing fully apart from them.

### 4.2 Longitudinal associations

The longitudinal findings show that mental wellbeing is characterised by substantial temporal stability, as levels of wellbeing were primarily related to their own prior levels rather than to changes in other measured variables. Similar stability was observed for academic stressors (workload, pressure, and time restraints), academic self-perceptions, coping styles, and social variables such as social support and social network satisfaction, suggesting that these constructs follow relatively continuous trajectories over the academic year. Associations across these variables were generally small, indicating that changes in academic stressors, coping, or social factors were only modestly related to subsequent changes in mental wellbeing. The association between social support at T1 and social network satisfaction at T2 suggests that perceived support may play a role in how students evaluate their social relationships over time. These findings indicate that mental wellbeing is not strongly influenced by short-term changes in academic stressors, coping styles, or social factors, but develops gradually over time. This pattern is consistent with findings from the systematic review and meta-analysis by du Toit et al. (2022), which shows that mental wellbeing demonstrates strong continuity and that individual predictors typically have small effect sizes, and that their influence tends to weaken as the time between measurements increases. The review also highlights that wellbeing is shaped by a range of personal and relational factors rather than a single dominant determinant. Together, these findings suggest that changes in student mental wellbeing are likely to occur gradually and cumulatively, implying that interventions may be most effective when implemented early, sustained over time, and embedded within the broader context in which students study and interact.

### 4.3 Methodological reflection and limitations

Several methodological considerations are important when interpreting these findings. Although the network approach offers a valuable descriptive framework for examining how multiple factors co-occur within a system, the cross-sectional network does not provide evidence for causal relationships and should therefore be interpreted as a representation of conditional associations rather than directional processes. The visual impression of layering within the network resonates with a socio-ecological interpretation, yet the absence of clear modularity in formal analyses combined with limited stability suggests that network methodology may not capture the hierarchical structuring implied by socio-ecological models. Instead of identifying distinct layers, the network approach primarily captures how influences from different socio-ecological levels are interrelated within one integrated structure, highlighting connections across levels rather than clearly outlining them. At the same time, by focusing on node-level centrality indices, the present study remained closely aligned with its aim of exploring the relative positioning of individual constructs within the broader network. Network-level measures, such as centralization, were therefore not central to the present research aim, as they address a different aspect of network structure: the extent to which the network as a whole is organized around one or a few dominant nodes. Such measures could add insight in future research by characterizing the overall organization of the network, rather than the position of individual constructs within it (Freeman, 1978).

The stability analyses further underscore the need for caution. When the network was re-estimated in bootstrap samples, several edge estimates showed considerable uncertainty. This means that some connections between variables may depend partly on the specific sample and should therefore be interpreted with caution. Although the sample size was considered sufficient for the exploratory purpose of this 14-node network analysis, limited power or precision for detecting and stabilizing weaker associations cannot be fully ruled out, because weak edges generally require larger samples to be estimated reliably. Centrality results should therefore be interpreted within this context: only strength and expected influence reached sufficient stability for descriptive interpretation, while betweenness and closeness were too unstable to interpret. This highlights both the potential and the constraints of applying centrality metrics in this research field, where constructs are often conceptually overlapping and measured at similar levels of abstraction, making it difficult to draw firm conclusions about the relative importance of individual variables.

The longitudinal analyses, although methodologically robust through the use of regularized regression, were based on a reduced sample due to attrition, which may have limited the detection of smaller cross-domain associations. At the same time, the conservative nature of LASSO penalization implies that only the most consistent relationships were retained, meaning that some meaningful but weaker associations may not have been captured. All variables were assessed using self-report measures, which may introduce shared method variance and reflect subjective perceptions rather than objective conditions. In addition, the study was conducted within a single educational context, which may limit the generalizability of the findings to other student populations or institutional settings.

Taken together, these considerations suggest that the application of a network analysis provides a nuanced but primarily exploratory insight into how academic, social, and coping-related factors are positioned in relation to mental wellbeing. At the same time, the findings indicate that the network approach has certain limitations in this context. When associations are relatively weak, constructs overlap conceptually, and parameter stability is limited, it may be more difficult to identify clear structures or to distinguish between variables. Future research could build on these insights by examining similar network configurations in other educational contexts and by incorporating additional measurement waves to better capture how interactions between these constructs evolve over time. Exploring how the positioning of social support and coping processes can inform the design of system-informed interventions may further contribute to strengthening student mental wellbeing in practice. In addition, future studies could move beyond purely descriptive network approaches by examining whether the observed patterns reflect more stable clusters or underlying dimensions, for example by combining network analysis with structural equation modelling or latent variable approaches. Such approaches may help to disentangle shared variance between closely related constructs and provide a clearer understanding of how academic, social, and coping-related factors are organised within the system.

## 5 Conclusion

By bringing together factors from different levels of the socio-ecological model, this study provides an exploratory understanding with a network approach of how academic pressures, social environments, and coping processes are positioned in relation to student mental wellbeing. The network showed that direct associations between variables were generally weak and largely confined between conceptually related variables, suggesting that variables tend to co-occur in related patterns rather than exert strong, independent relationships with mental wellbeing. Mental wellbeing and social support occupy the most central positions in the estimated network, indicating that they are most strongly embedded in the broader system of student experiences. Academic self-perceptions is positioned in close relation to academic strain variables, suggesting a potential counterbalancing role within these patterns. In addition, emotion-focused coping occupies a notable position, as it is connected to both more adaptive and more avoidant coping responses, indicating a potential bridging role within coping processes. Together with the longitudinal findings, which showed that mental wellbeing was primarily associated with its own prior level, the results suggest that wellbeing develops within a relatively stable system in which multiple experiences co-occur, while short-term changes in individual factors are only modestly related to changes in mental wellbeing over time.

## CRediT authorship contribution statement

**Sybren Slimmen:** Writing – original draft. **Olaf Timmermans:** Writing – original draft. **Lilian Lechner:** Writing – original draft. **Peter Verboon:** Writing – original draft. **Anke Oenema:** Writing – original draft.

## Ethical statement

This study was approved by the ethical review board of the Open University, the Netherlands (reference number: CETO_RP459/U202309407). All participants provided informed consent prior to participation. Participation was voluntary, and data were collected anonymously through an online survey platform in accordance with applicable data protection regulations. The study was preregistered in the Open Science Framework (OSF; https://doi.org/10.17605/OSF.IO/BUCRM).

## Declaration of competing interest

The authors declare that they have no known competing financial interests or personal relationships that could have appeared to influence the work reported in this paper.

## Data Availability

Data will be made available on request.
