# Supplementary material for: Student mental wellbeing as an interconnected system: positioning academic, social, and coping processes
Source: SSM Popul Health. 2026 Jun 8;35:101936. doi: 10.1016/j.ssmph.2026.101936 (PMC13292374; doi:10.1016/j.ssmph.2026.101936)
Supplement: Multimedia component 1 [file mmc1.docx]

**Supplementary File**

Title of article: *Student mental wellbeing as an interconnected system: positioning academic, social, and coping processes*

This supplementary file provides additional methodological details and visualizations to support the interpretation of the network and longitudinal analyses presented in the main manuscript. It includes results from bootstrap procedures used to assess the accuracy and stability of the estimated network, as well as additional insight into the contribution of individual edges and nodes.

Figure A presents nonparametric bootstrap confidence intervals for the estimated edge weights, allowing for an evaluation of the robustness and precision of the observed associations. Figure B shows the stability of centrality indices based on a case-dropping bootstrap procedure, providing insight into the reliability of node-level metrics such as strength, closeness, and betweenness. Figure C offers a matrix visualization of node strength and the corresponding non-zero edges retained after regularization, illustrating how individual associations contribute to the overall positioning of variables within the network.

Together, these materials are intended to support a more detailed and transparent evaluation of the network structure and to contextualize the interpretation of centrality and edge estimates reported in the main text.

**Supplementary Methodological Information**

**Bootstrap analysis**

Non-parametric bootstrapping (1000 samples) was used to obtain confidence intervals around edge weights. Centrality stability was examined using a case-dropping bootstrap procedure and quantified by the correlation-stability (CS) coefficient. Following recommendations by Epskamp et al. (2018), only centrality metrics with a CS coefficient above .25 were considered sufficiently stable for interpretation.

**Longitudinal associations**

To examine longitudinal associations between the study variables across measurement occasions, we applied a penalized regression approach using the Least Absolute Shrinkage and Selection Operator (LASSO) implemented in R (RStudio) with the glmnet package (Hastie & Qian, 2014). This technique is particularly suitable in settings with a relatively large number of potentially correlated predictors relative to the sample size, as it reduces the risk of overfitting and facilitates variable selection by shrinking small regression coefficients exactly to zero (Hastie & Qian, 2014; McNeish, 2015). In the present analysis, variables measured at T1 were entered simultaneously as predictors of corresponding and non-corresponding variables measured at T2. The LASSO penalty imposes an ℓ₁ constraint on the regression coefficients, resulting in sparse models that retain only the most relevant associations while excluding weak or redundant predictors. To examine how variables measured at T1 were related to outcomes at T2, we used a stepwise procedure that determines which predictors should be retained in the analysis. This procedure repeatedly tests different versions of the model on subsets of the data to prevent overfitting. A conservative setting was chosen using the one–standard-error rule (λ.1se), which favours simpler solutions and retains only predictors that show consistent associations across subsets of the data (Hastie & Qian, 2014). Coefficients that remained non-zero at the selected penalty level were interpreted as longitudinal associations between variables across time points. Following recommendations by van Erp et al. (2019), the results were interpreted descriptively, highlighting associations that remained after shrinkage and excluding weaker relationships.

**Figure A**

*Nonparametric bootstrap confidence intervals for edge weights in the EBICglasso-estimated network*
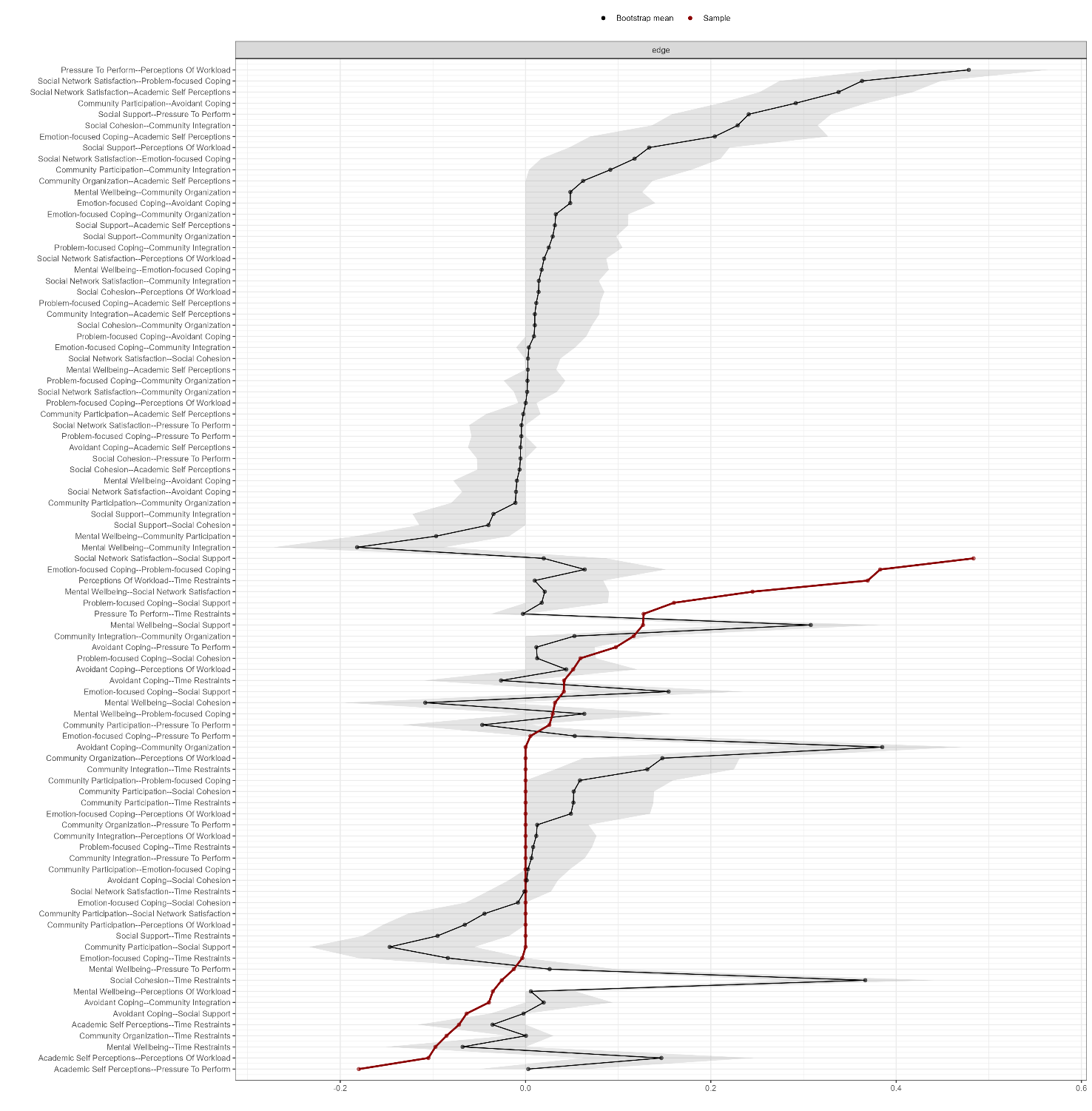


**Note**. For each edge, the point estimate obtained from the original sample is shown in red, while the black dots represent the mean edge weight across 1,000 bootstrap samples. The shaded gray areas indicate the nonparametric bootstrap confidence intervals. Edges are ordered by their estimated strength. Confidence intervals overlapping zero indicate edges whose estimated weight is unstable and should be interpreted with caution, whereas narrower intervals away from zero indicate more stable edge estimates.

**Figure B**

*Stability of centrality indices (strength, closeness, and betweenness) assessed using a case-dropping bootstrap procedure*
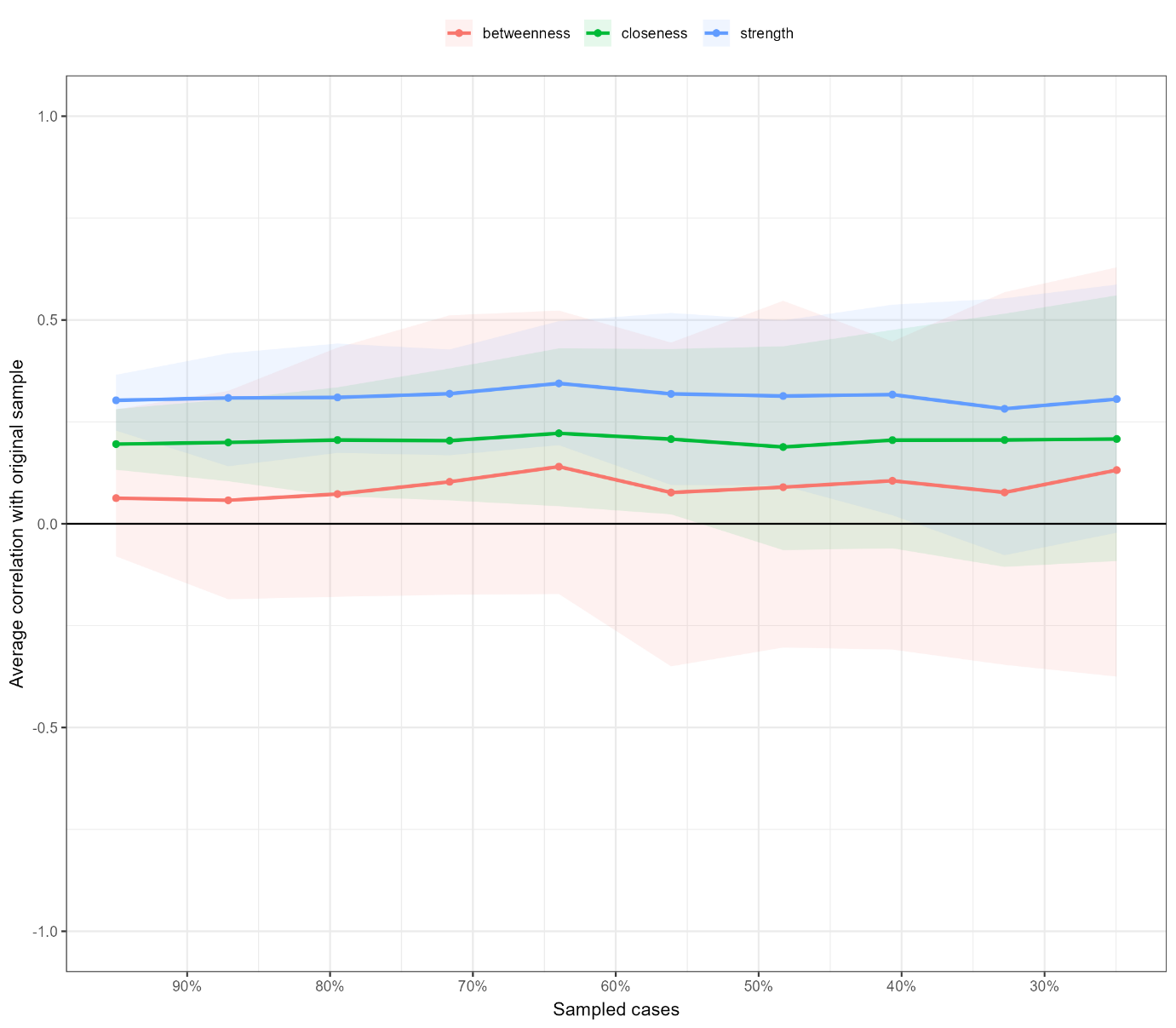


**Note.** The figure shows the correlation between centrality estimates obtained from subsets of the data and those from the original sample, based on 1,000 case-dropping resamples. The x-axis indicates the proportion of cases retained, while the y-axis represents the average correlation with the original centrality estimates. Shaded areas reflect variability across resamples.

**Figure C**

*Matrix visualization of node strength and contributing edges in the EBICglasso-estimated network*
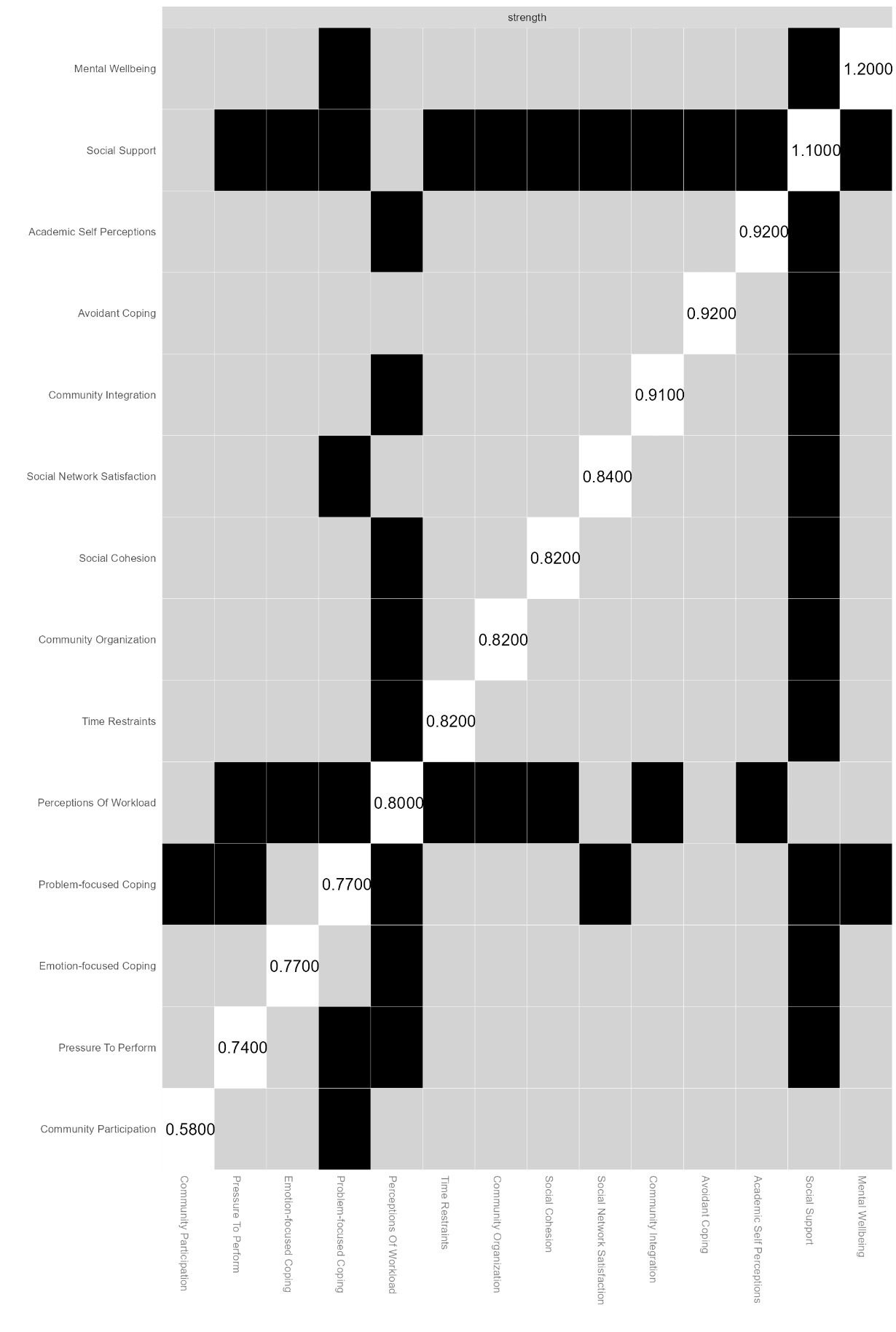


**Note.** Black cells indicate non-zero edges retained after regularization, gray cells indicate edges shrunk to zero, and diagonal values represent node strength.

**References**

Epskamp, S., Borsboom, D., & Fried, E. I. (2018). Estimating psychological networks and their accuracy: A tutorial paper. *Behavior Research Methods*, *50*(1), 195-212. <https://doi.org/10.3758/s13428-017-0862-1>

Hastie, T., & Qian, J. (2014). Glmnet vignette. *Retrieved June*, *9*(2016), 1-30.

McNeish, D. M. (2015). Using lasso for predictor selection and to assuage overfitting: A method long overlooked in behavioral sciences. *Multivariate behavioral research*, *50*(5), 471-484.

van Erp, S., Oberski, D. L., & Mulder, J. (2019). Shrinkage priors for Bayesian penalized regression. *Journal of Mathematical Psychology*, *89*, 31-50. <https://doi.org/https://doi.org/10.1016/j.jmp.2018.12.004>
